# The cost-effectiveness of exercise-based cardiac rehabilitation: a systematic review of the characteristics and methodological quality of published literature

**DOI:** 10.1186/s13561-017-0173-3

**Published:** 2017-10-19

**Authors:** Katherine Edwards, Natasha Jones, Julia Newton, Charlie Foster, Andrew Judge, Kate Jackson, Nigel K. Arden, Rafael Pinedo-Villanueva

**Affiliations:** 1 Nuffield Department of Orthopaedics, Rheumatology and Musculoskeletal Sciences, University of Oxford, Oxford, UK; 2 Faculty of Sport and Exercise Medicine, Edinburgh, UK; 3 Nuffield Orthopaedic Centre, Oxford, UK; 4 Arthritis Research UK Centre for Sport, Exercise and Osteoarthritis, University of Oxford, Oxford, UK; 5 Nuffield Department of Population Health, University of Oxford, Oxford, UK; 6 MRC Lifecourse Epidemiological Unit, University of Southampton, Southampton, UK

**Keywords:** Cardiac rehabilitation, Cost effectiveness, Economic evaluation, Exercise, Cheers

## Abstract

**Aim:**

This descriptive review aimed to assess the characteristics and methodological quality of economic evaluations of cardiac rehabilitation (CR) programs according to updated economic guidelines for healthcare interventions. Recommendations will be made to inform future research addressing the impact of a physical exercise component on cost-effectiveness.

**Methods:**

Electronic databases were searched for economic evaluations of exercise-based CR programs published in English between 2000 and 2014. The Consolidated Health Economic Evaluation Reporting Standards (CHEERS) statement was used to review the methodological quality of included economic evaluations.

**Results:**

Fifteen economic evaluations met the review inclusion criteria. Assessed study characteristics exhibited wide variability, particularly in their economic perspective, time horizon, setting, comparators and included costs, with significant heterogeneity in exercise dose across interventions. Ten evaluations were based on randomised controlled trials (RCTs) spanning 6–24 months but often with weak or inconclusive results; two were modelling studies; and the final three utilised longer time horizons of 3.5–5 years from which findings suggest that long-term exercise-based CR results in lower costs, reduced hospitalisations and a longer cumulative patient lifetime. None of the 15 articles met all the CHEERS quality criteria, with the majority either fully or partially meeting a selection of the assessed variables.

**Conclusion:**

Evidence exists supporting the cost-effectiveness of exercise-based CR for cardiovascular disease patients. However, variability in CR program delivery and weak consistency between study perspective and design limits study comparability and therefore the accumulation of evidence in support of a particular exercise regime. The generalisability of study findings was limited due to the exclusion of patients with comorbidities as would typically be found in a real-world setting. The use of longer time-horizons would be more comparable with a chronic condition and enable economic assessments of the long-term effects of CR. As none of the articles met recent reporting standards for the economic assessment of healthcare interventions, it is recommended that future studies adhere to such guidelines.

## Introduction

The global individual and economic burden of cardiovascular disease demands continual innovation of prevention and treatment strategies for effective patient management [1, 2]. Competition between interventions is accentuated by increasing financial constraints on healthcare resources [2]. Economic evaluations provide a useful comparative approach for effective and efficient policy and decision-making considering both costs and consequences on patient outcomes [3, 4].

Cardiac rehabilitation (CR) programs are a standard part of cardiac patient care [5]. Exercise is recognised as a core component of CR and is provided alone, or within a multidisciplinary program combining risk factor management, behaviour modification and psychosocial support [6, 7].

For cardiac patients, the cost-effectiveness of CR compared to standard care has been estimated to cost between USD$2000–$28,000 per life-year gained or leading to increased health-related quality of life (HRQL) at a cost of USD$700–$16,000 per quality-adjusted life-year (QALY) gained [3].

With new CR service-delivery models emerging and healthcare resources becoming more limited, it is timely to reassess the cost-effectiveness of CR-services. Also with the recent development of updated standards for economic evaluations of healthcare interventions, it is necessary to bring the findings of previous reviews [3, 5] into context with these guidelines as to provide a platform for future studies looking at the cost-effectiveness of CR services to build upon. With that in mind, this systematic review aims to understand how economic evaluations of exercise-based CR are conducted with the following objectives: (i) to review the characteristics of published economic evaluations of exercise-based CR with exercise as the primary outcome of interest; (ii) to evaluate the methodological quality of these CR economic evaluations using the Consolidated Health Economic Evaluation Reporting Standards (CHEERS) checklist [8] and (iii) to make recommendations for future economic evaluations of CR services. This descriptive study will inform the quality of future research addressing the cost-effectiveness of exercise rehabilitation interventions.

## Methods

### Economic evaluation

Economic evaluations consist of partial or full analyses [3]. Partial evaluations assess either costs or consequences of multiple interventions or both costs and consequences of a single intervention. Full evaluations examine both costs and consequences of multiple interventions [3]. This review includes all forms of full economic evaluations, i.e. cost-effectiveness, cost-benefit or cost-utility analyses.

### Literature search strategy

The “PICO” statement was used to define the search criteria for the review and identify the specifics of the patient population, intervention and the types of studies to be evaluated. Electronic databases (Medline, Embase, HTA, DARE, NHS EED and the Cochrane Library) were searched for all (UK and worldwide) full economic evaluations of CR published in English between 2000 and 2014. The following text-word terms and MeSH headings were used: cost, cost analysis, cost benefit, cost effectiveness, cost minimisation, cost utility, economic assessment, economic evaluation, health economics and cardiac rehabilitation (Appendix 1). Hand searches of bibliographies identified additional publications of which any date was included. Hand searching references of rejected publications also ensured that significant publications of relevance to the field were not missed. Grey literature was not included, but this is unlikely to have any significant effects on publication bias as most economic evaluations are published or cited in scientific or economic journals and will have been picked up through the extensive online literature search.

### Selection criteria

A study was considered if it met all the following inclusion criteria:

1. Adult patients with heart disease/failure who have undergone myocardial infarction (MI) or revascularisation (percutaneous transluminal coronary angioplasty (PTCA) or coronary artery bypass grafting (CABG)) and participated in a CR program
2. Intervention includes an exercise-based CR program with follow-up
3. A full-economic evaluation

### Data extraction

Two reviewers (KE and RPV) independently selected eligible publications. Disagreement between reviewers was resolved through direct consultation. Data extraction was carried out by a single reviewer (KE) and checked by RPV. Data was extracted from eligible publications on the following items from CHEERS [8]: target population and subgroups, setting and location, study perspective, comparators, time horizon, choice of health outcomes, measurement of effectiveness, measurement and valuation of preference-based outcomes, estimating resources and costs, choice of model, currency, price date and conversion, characterising uncertainty and characterising heterogeneity. Additional data was extracted on study design, sample demographics, exercise dose, frequency and duration of follow-up, included costs, chronic multimorbidity and findings.

### Quality assessment

The methodological quality of included evaluations was assessed using CHEERS guidelines for each of the data items extracted [8]. In addition to reporting whether individual studies meet the criteria for each of the data items, their subsequent effects on the study results (such as uncertainty) are examined. The guideline criteria assess specific design elements of economic evaluations for healthcare interventions. CHEERS is not known to have previously been used to assess economic evaluations of exercise-based CR interventions.

## Results

### Synthesis of evidence

**Fig. 1:**
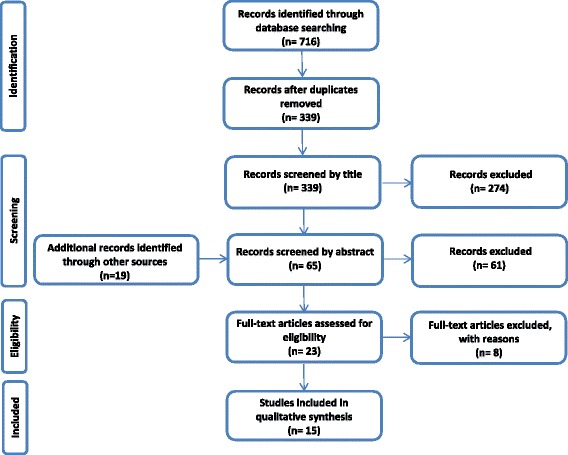
Flow of Review Selection Process

The search strategy retrieved 716 citations, 23 qualified for full-text review and eight were further excluded. Excluded were two literature reviews, one abstract, two which only reported on study designs, one reporting on an already included study, and two which were not full-economic evaluations. Southard et al. [9] is not designed as a full-economic evaluation and structured as a descriptive assessment, but it was included as it examines both health outcomes and costs between two CR interventions. Figure 1 illustrates the review selection process.

## Descriptive analysis

Descriptive characteristics of the 15 included economic evaluations are provided in Appendix 2.

### Dates, study design, perspective, time horizon and location

The 15 included articles were published between 1991 and 2008, with twelve (80%) being based in the USA or in Europe. Ten studies (67%) were based on randomised clinical trials (RCTs) with time horizons ranging from 6 to 24 months. Twelve (80%) of the studies adopted a cost-effectiveness or cost-utility approach, with the remaining three taking the cost-benefit approach. Eight (53%) of the included studies evaluated costs from the perspective of the healthcare system, with three of those additionally considering patient-borne costs (Table 1).

**Table 1:** Study design, location and study perspective of included studies

Study Design		Study Location	Study Perspective
RCTs	10 (67%)	UK	Healthcare system and patient
			Societal
		USA	Healthcare system Insurer
		Canada	Healthcare system Healthcare system Healthcare system and patient
		Australia	Healthcare system Healthcare system and patient
		Hong Kong	Patient and provider
Non-RCTs	2 (13%)	Belgium	Healthcare system
		Sweden	Societal
Modelling Studies	2 (13%)	USA	Societal Patient or Payer
Cohort Studies	1 (7%)	USA	Insurer

### Program setting, comparators and target population

The comparisons assessed by the selected studies varied greatly. They were undertaken based on various features of the intervention such as where the CR program took place, how exercise was incorporated into the program, or even how the exercise component was delivered.

Taylor et al. [10] and Jolly et al. [11] compared hospital- vs. home- exercise-based CR. Five studies compared hospital-based CR interventions including exercise: Briffa et al. [12] against conventional care where it is unclear if exercise is a component; Levin et al. [13] with standard care and no provision of regular exercise; Dendale et al. [14] with no CR; Hall et al. [15] with no formal CR but provision of a home-walking program; and Ades et al. [16] devised a model based primarily on hospital CR programs that were compared to light/no exercise.

Four studies were at a rehabilitation facility: Papadakis et al. [2] and Reid et al. [17] were based on the same trial and evaluated service-delivery differences between a 3 and 12-month exercise-based CR program, whilst Carlson et al. [18] compared a traditional exercise-based program to one with tapered exercise sessions, and Yu et al. [19] compared an exercise-based CR program to conventional therapy without exercise.

Southard et al. [9] compared a home-based internet program for monitoring patient exercise to usual care, where it is unclear if exercise is a component.

In three studies, the setting of the exercise was unclear: the modelling study by Spronk et al. [20] compared three exercise-based CR strategies; Huang et al. [21] exercise-based CR to no-CR; and Oldridge et al. [4] exercise-based CR against usual care, but did not specify if exercise was a component of that care. Table 2 compares the interventions of each study according to program setting and exercise duration.

**Table 2:** Interventions compared by setting and duration of exercise

	Duration of Exercise									
	No Exercise	1–2 weeks	6 weeks	8 weeks	9 weeks	3 months	6 months	12 months	24 months	Un-specified
Jolly et al. (2007) [11]			Home Hosp							
			Home	Hosp						
			Home		Hosp					
			Home			Hosp				
Yu et al. (2004) [19]	X								Rehab^a^	
Papadakis et al. (2007) [2]						Rehab^a^		Rehab		
Briffa et al. (2005) [12]			Hosp							X
Hall et al. (2002) [15]		Home	Hosp							
Taylor et al. (2007) [10]			Home		Hosp (8–10)					
Dendale et al. (2008) [14]	X					Hosp^b^				
Reid et al. (2005) [17]						Rehab		Rehab		
Southard et al. (2003) [9]							Home^b^			X
Carlson et al. (2000) [18]							Rehab Rehab^a^			
Levin et al. (1991) [13]	X								Hosp^b^	
Oldridge et al. (1993) [4]				Un^a^						X

(Setting of Exercise); Hosp = Hospital-based exercise intervention; Home = Home-based exercise intervention; Rehab = Exercise intervention based at rehabilitation centre; No Ex = No exercise intervention; Un = Unclear (Significance Level); ^a^ statistically significant differences were identified for either cost or health outcomes in favour of specified intervention arm, ^b^ statistically significant differences were identified in both cost and health outcomes in favour of specified intervention arm

In twelve studies the target population included MI patients, one included only PCI patients [14], another only CABG patients [21], and one specified only low-risk cardiac patients who experienced cardiovascular surgery or an event (MI, PCI or CABG) [18].

### Sample demographics, Subgroups and Comorbidities

Excluding the two modelling studies, the cohort study employed the largest sample at 4324 patients [21]. In the remaining trials, sample size ranged from 80 to 525 patients at initial recruitment; the majority were males who in most cases accounted for over 72% of the overall sample, with mean age ranging from 53 to 65 years.

Two studies conducted subgroup analyses, each looking at different variables (e.g. age, sex, BMI, cardiac risk, reason for referral) and extent of, if any, their effect on costs [2, 21].

Nine studies made no reference to comorbidities. Three excluded patients with major [10], life threatening or symptomatic comorbidities [14], or where program participation was prevented [11]. Two documented comorbidities, one reporting only cardiovascular-related comorbidities [12, 21]. One reported addressing co-morbidities but provided no details [17]. No study reported analysing data using comorbidities to stratify patients.

### Dose of exercise

Excluding the two modelling studies [16, 20] and the cohort study [21], the exercise dose of interventions was assessed using FITT (frequency, intensity, time and type). Southard et al. [9] is classified as patient-dependent for all variables due to the intervention being home-based. Appendix 3 offers a detailed description of exercise dose by intervention within each selected study.

#### Frequency

The remaining 11 evaluations reported patient exercise frequency, with one referencing the original publication [4, 22]. The frequency of provision for supervised exercise sessions ranged from once-weekly [10] up to four-times weekly [15], with the majority providing twice-weekly sessions [2, 4, 13, 17, 19]. One combined data from three hospital-based CR programs where the frequency of exercise sessions was once-weekly, twice-weekly and tapered [11].

#### Intensity

Eight studies reported patient exercise intensity, five directly [11, 14, 17–19] and three through original trial publications [2, 4, 13, 22, 23]. The threshold for exercise intensity varied between these studies with the majority aiming for between 60 and 85% maximum heart rate capacity [4, 11, 18, 19]. Three did not report patient exercise intensity [10, 12, 15].

#### Time

Seven evaluations directly reported the duration of patient supervised exercise [11–14, 17–19], while two referred to original trial publications [2, 4, 22]. In these studies, exercise duration ranged from 30 to 120 min. Two did not report exercise duration on their respective CR programs [10, 15].

#### Type

Nine evaluations directly reported, with varying detail, on the type of exercise undertaken by CR program patients [4, 11–15, 17–19], while two referred to original trial publications [2, 10, 24]. The majority involved aerobic exercise training with some specifying the activities involved (e.g. running, cycling, rowing, and circuits). Two stated only that it was a low-level exercise program [4, 15], and one that exercise was ‘consistent with guidelines for patients with CAD’ (coronary artery disease) [17].

### Health outcomes and measures of effectiveness

Included studies assessed a variety of health outcomes: (i) HRQL, (ii) cardiovascular health, and (iii) survival. Three were cost-benefit analyses and assessed the monetary equivalent of cardiovascular events [9, 14] or total cost over the study period [13]. Eight evaluated HRQL outcomes: seven using the QALY as an effectiveness measure and either the EQ-5D (using the UK value set tariff) [10, 11], time-trade off scores [3, 4, 19, 20], or UBQ-H scores [12] as preference-based outcomes, while one used a Quality-of-Life score derived from the Health Measurement Questionnaire as an effectiveness measure with no preference-based outcome [15]. Two evaluations assessed cardiovascular health outcomes using factors including peak oxygen consumption, cholesterol levels or kilojoules of activity-related energy as measures of effectiveness [17, 18]. Two looked at survival, with Years-of-Life-Saved (YLS) as the effectiveness measure with no preference-based outcome [16, 21] (Appendix 4).

## Economic analysis

The following section reports on features of the health economic analyses. Details are provided in Appendix 5.

### Costs

All evaluations considered direct medical costs relating to CR service-provision. Seven considered patient costs, including direct expenses [12], travel [4, 10, 11, 15, 20], time [4, 13, 20], equipment [4, 10] or childcare expenses [4]. One also considered costs associated with employee productivity loss from sick leave or early retirement [13].

### Source of costs

Selected studies estimated costs from a combination of sources, including hospital-derived data [2, 15, 20], local/national publications [2, 12, 19], or health insurance companies [13, 17, 21]. One used published data from results of previous RCTs [16]. One did not report the source of their cost data [18].

### Currency, price date and conversion

The majority of studies reported costs in United States dollars (USD$), with three having converted from Canadian dollars (CAN$) using a set exchange rate [2, 4, 17]. Two reported costs in British Pounds (£) [10, 11], two in Australian dollars (AUD$) [12, 15], one in Euros (€) [14], and one in Swedish Kroner (SEK) [13]. Two present costs in unspecified dollars, assumed to be USD$ based on the authors’ affiliations [9].

### Uncertainty

Nine evaluations characterised the uncertainty around their results: three applied one-way sensitivity analysis [11, 13, 16], Spronk et al. [20] used a combination of one-way, two-way, multi-way and probabilistic sensitivity analyses, and both Briffa et al. [12] and Taylor et al. [10] combined sensitivity analysis and bootstrapping methods. Huang et al. [21] used bootstrapping methods alone, Papadakis et al. [2] combined this technique with a cost-effectiveness acceptability curve (CEAC), and Oldridge et al. [4] used scenario analysis to calculate the plausible range of costs using the minimal, mean and maximum estimates of direct medical costs per patient.

### Heterogeneity

Only two evaluations performed subgroup analysis allowing the presentation of results by patient subgroups: Huang et al. [21] reported Medicare expenditure and survival by age, sex, race, number of cardiovascular conditions, diagnosis of chronic obstructive pulmonary disease (COPD), Medicare coverage, higher serum albumin, primary diagnosis of diabetes, AMI before CABG and propensity for receiving CR, while Papadakis et al. [2] provided mean incremental costs, QALYS gained and incremental cost-effectiveness ratio (ICER) by cardiac risk level, risk of disease progression, reason for referral and sex.

### Choice of model

The review included two modelling studies: Spronk et al. [20] employed a Markov model to compare the cost-effectiveness of three exercise-based CR strategies, and Ades et al. [16] devised a statistical model to calculate cost-effectiveness in Years-of-Life-Saved.

## Findings

### Hospital-based CR

Five evaluations compared hospital-based exercise interventions: one to ‘standard’ care [13], one to ‘conventional’ care [12], two with no-CR [14, 15], and one to light/no exercise [16]. Against standard care, hospital-based CR was highly cost-saving over 5 years, with lower direct healthcare costs (3910SEK/€409), fewer cardiovascular-related rehospitalisation’s (0.6 events/patient, non-significant) and less time receiving in-hospital treatment (5.4 days, *p* < 0.05) [13]. Compared to conventional care, survival advantages are reported for hospital-CR patients where rehabilitation costs of AUD$631(€471)/patient were offset by reduced follow-up costs of AUD$236(€156)/patient and a non-significant gain in quality-of-life up to a year [12]. Against no-CR, one study found hospital-based CR cost €636 less/patient with a reduced number of cardiovascular-related events (0.59 events/patient) [14]. Another estimated CR program cost at AUD$300(€198) but, with non-significant differences between costs and health outcomes compared to no-CR patients, suggests savings may be made by targeting rehabilitation to high-risk individuals [15]. Over 15-years, hospital-based CR is reportedly highly cost-effective and compares favourably to alternative treatments (e.g. thrombolytic therapy, cholesterol-lowering drugs) with a net incremental cost of USD$430 ($1280 vs $850)/€357 (€1063 vs €706), a discounted incremental life expectancy of 0.202 years and an ICER of USD$4950(€4111)/YLS [16].

### Home-based CR

One study used a home-based internet intervention to monitor patient activity and as a platform for guidance and interaction [9]. Over usual care, this had net cost-savings of USD$965(€801)/patient and an 11.6% reduction in major cardiovascular events [9].

### Hospital- versus home-based CR

Two evaluations examined cost-effectiveness between hospital and home-based CR programs including exercise [10, 11]. Neither found significant differences for costs or health outcomes between patient groups. Taylor et al. [10] found home-based CR had a lower mean cost/patient due to reduced personnel costs (UK£30, 95% CI -£45 to -£12/€32, 95% CI -€48 to -€13), but was associated with greater healthcare costs (UK£78, 95% CI, −£1102 to £1191/€84, 95% CI -€1185 to €1281). Jolly et al. [11] found home-based CR had higher direct rehabilitation costs to the health service (UK£41, 95% CI £26 to £55/€44, 95% CI €28 to €59), even after including patient costs to the hospital-based arm. Each found a non-significantly worse difference in health outcomes for home-based patients with mean QALY differences of −0.022 (95% CI -0.072 to −0.028) [11] or −0.06 (SD, −0.15 to 0.02) [10] between interventions.

### Centre-based CR

Four studies were based at a rehabilitation facility: two compared 3 and 12-month exercise-based interventions [2, 17], one a 6-month exercise program to one with tapered sessions [18], and another exercise-based CR to conventional care without exercise [19]. A 3-month CR program cost USD$135(€112) less than a 12-month program, with non-significant differences for exercise-related variables, cardiac risk factors and HRQL [17]. With the same trial, another study showed the 3-month program had an incremental gain of 0.009 QALYs (95% CI 0.004–0.013) and no significant cost differences to a 12-month program [2]. Sub-group analysis showed the 3-month program was dominant for patients with high-risk of disease progression while the 12-month program was preferential for PCI patients, suggesting triaging patients may improve cost-effectiveness [2]. Another study showed 6-month hospital-based CR with tapered exercise sessions was USD$738(€612)/patient cheaper than one with consistent thrice-weekly sessions, but with no significant differences between groups in outcome measures [18]. Against conventional care without exercise, CR was USD$416(€345)/patient cheaper with a 0.6 QALY gain after 2-years, but non-significantly [19].

Three studies do not explicitly report exercise setting. One compared three exercise-based CR strategies (CR only, diagnostic work up for revascularisation before CR or after CR failure), of which the latter was the most favourable with a non-significant gain of 0.03 QALYs and an ICER of USD$44,251(€36,728)/QALY over a patient lifetime compared to CR only [20]. Another study compared exercise-based CR to no-CR finding over 3.5-years finding CR highly cost-effective, associated with a longer cumulative lifetime (76 days, 95% CI 22–129 days) and ICER of USD$13,887(€11,526)/YLS [21]. The remaining study concluded exercise-based CR was an efficient use of healthcare resources with a best incremental cost of USD$480(€398) and mean QALY gain of 0.052 leading to an expected ICER of USD$9200(€7638)/QALY (range USD$2300 to $182,800/€1910 to €151,769) over usual care [4].

### Quality assessment

None of the 15 articles met all the CHEERS criteria for included variables (Table 3). All 15 met the reporting recommendations for study perspective and measure of effectiveness. A mixture of studies fully or partially met criteria for reporting target population, setting/location, comparators, estimating resources and costs, and currency. Nine studies characterised the uncertainty of their results, of which only seven fully met the criteria. Two studies fully met the criteria for reporting choice of health outcomes as others did not specify the relevance of those chosen [2, 10]. Two studies performed sub-group analysis, with only one reporting between group variation for incremental costs, QALYs and ICER values [2, 21]. None fully met the criteria for time horizon, as none stated why that used was appropriate. CHEERS assessment was based on the content of individual articles only and not in conjunction with overlapping publications which may have contained relevant information.

**Table 3:** Quality assessment of included economic evaluations

	Target Population/ Subgroups	Setting/Location	Study Perspective	Comparators	Time Horizon	Choice of Health Outcomes	Measure of Effectiveness	Measurement & Validation of Preference-based outcomes	Estimating Resources and Costs	Currency, price, Date and Conversion	Choice of Model	Characterising Uncertainty	Characterising Heterogeneity
Jolly et al. (2007) [11]	Y	Y	Y	Y	O	O	Y	Y	Y	Y	N/A	O	N
Yu et al. (2004) [19]	Y	Y	Y	Y	O	O	Y	Y	O	O	N/A	N	N
Papadakis et al. (2007) [2]	Y	O	Y	Y	O	Y	Y	O	Y	Y	N/A	Y	Y
Spronk et al. (2008) [20]	Y	O	Y	Y	O	O	Y	Y	Y	Y	O	Y	N
Briffa et al. (2005) [12]	Y	Y	Y	O	O	O	Y	Y	Y	Y	N/A	O	N
Hall et al. (2002) [15]	Y	Y	Y	Y	O	O	Y	N/A	O	Y	N/A	O	N
Taylor et al. (2007) [10]	O	Y	Y	Y	O	Y	Y	O	Y	Y	N/A	Y	N
Dendale et al. (2008) [14]	Y	Y	Y	Y	O	O	Y	N/A	Y	Y	N/A	N	N
Reid et al. (2005) [17]	Y	Y	Y	Y	O	O	Y	N/A	O	Y	N/A	N	N
Southard et al. (2003) [9]	Y	O	Y	O	O	O	Y	N/A	O	O	N/A	N	N
Huang et al. (2008) [21]	O	O	Y	Y	O	O	Y	N/A	Y	Y	N/A	Y	O
Carlson et al. (2000) [18]	Y	Y	Y	Y	O	O	Y	N/A	N	O	N/A	N	N
Levin et al. (1991) [13]	Y	Y	Y	Y	O	O	Y	N/A	O	Y	N/A	Y	N
Oldridge et al. (1993) [4]	Y	O	Y	O	O	O	Y	O	O	Y	N/A	Y	N
Ades et al. (1997) [16]	Y	Y	Y	Y	O	O	Y	N/A	Y	Y	O	Y	N

Y = yes, N = no, O = partial, N/A = not applicable

## Discussion

This review assessed how economic evaluations of exercise-based CR programs are conducted and evaluated their methodological quality against the recently published CHEERS guidelines for healthcare interventions [8]. Exercise was the primary outcome of interest in this review as it has proven health benefits [25] and is a principal component of CR services; other aspects of CR including psychological or educational interventions were not evaluated.

An extensive literature search identified 15 economic evaluations of exercise-based CR services. In consensus with previous reviews we identified wide variability amongst CR programs and service delivery [3, 5]. In this review, such variability was particularly evident in study perspective, time horizon, setting, comparators, included costs, and in exercise dose (FITT) between interventions. We critically appraised included evaluations against recently expanded and updated economic guidance, finding that none fully met the reporting criteria; while included studies predated development of this guidance, future studies may wish to adhere to these up-to-date standards [8].

As most evaluations (10) were RCTs, their meticulous patient selection process will question the wider generalisability of their findings. Comparatively other study types report higher proportions of males (60–89%) and greater CR uptake (64–72%). [25]. Patients in these RCTs were also younger than the average age distribution for CR participants (67 for men and 70 for women) [25]. The use of short time horizons (6–24 months) also seems incompatible with a chronic condition. Given the likelihood that patients registering with a controlled trial may be more inclined to adhere to exercise requirements, these elements suggest economic evaluations of exercise-based CR programs using RCT’s risk providing non-generalizable results.

Compared to RCT’s reporting non-conclusive or weak results, evaluations utilising studies with longer time horizons (3.5 – 5 years) suggest a long-term exercise-based CR program results in lower costs [13, 14], reduced hospitalisations [13, 14], and longer cumulative lifetime [21]. Longer follow-up times may allow for more benefits of the intervention to be accrued and suggest that interventions should be carried out with a long-expanding time horizon.

Despite a reported 60–70% of cardiac patients accessing CR services having comorbidities, these patients were largely absent from included studies. This has been recognised and it is estimated that 48% are deemed inappropriate for rehabilitation by their referrer [25]. In this review most studies failed to report co-morbidities or simply excluded such patients [11, 12]. The likely presence of comorbidities in the population, particularly in older individuals, questions generalisability of findings, and reflects a missed opportunity for their management.

This review identified extensive heterogeneity between studies in exercise dose (FITT) [25]. Session frequency ranged from once to four times weekly, exercise intensity was patient dependent or categorised in broad groups (low, moderate or high intensity), and exercise type often involved combinations of aerobic activity (e.g. walking, running, cycling, rowing, arm cranking, dumbbell or weight training). This reflects a lack of knowledge and absence of guidance on the most effective CR exercise program. Standardising CR would allow more accurate economic assessments, although risk eliminating the potential for more cost-effective results to be obtained from patient-dependent CR exercise regimes [18]. Alternatively, harmonising physical exercise dose into a common standard unit, such as the metabolic equivalent of tasks (METs), would allow for an effective comparison of the very diverse interventions found in the literature [26, 27].

Generic quality-of-life measures (i.e. QALY) allow a common measure across health conditions to facilitate healthcare resource allocation, but their broad scope fails to capture other health-related benefits outside the dimensions of the questionnaire (mobility, self-care, usual activities, pain/discomfort and anxiety/depression in the EQ-5D). The difficulty associated with measuring exercise is a challenge for such interventions and its effects has been captured elsewhere [28]. Exercise is known to have far-reaching benefits proven effective at reducing the disease burden of diabetes, osteoarthritis and cancer [25], however generic HRQL instruments (e.g. EQ-5D and the SF-36) are likely to be insensitive to detecting change brought about by exercise-based CR [25]. Using more specific outcome measures, such as the change in physical activity level or evaluating the psychology of exercise behaviour (e.g. BREQ questionnaire), will provide a more complete picture of the benefits produced by the interventions and avoid producing inaccurate and misleading cost-effectiveness results. Given many studies found non-significant differences in costs between interventions, differences in health outcomes have the capacity to be the main drivers of cost-effectiveness. Appropriate criteria to detect and measure health impact according to the specific study design must be applied. [29–31].

All studies incorporated direct CR medical costs into their evaluations, but lacked consistency in the types of costs included and would likely result in two evaluations of the same clinical study reporting different cost-effectiveness results. Use of standardised cost categories consistent with the study aims, perspective and nature of exercise is recommended. For exercise-based CR, the cost-savings attributable to reduced cardiovascular events and potential reduction of general healthcare resource use should be reported. Given that several studies found a non-significant difference in health outcomes between interventions, costs are a potential driving force behind cost-effectiveness.

Few studies reported statistically significant evidence in both costs and effects for CR (Table 2). These were predominantly cost-benefit analyses comparing exercise-based CR to no exercise or where the use of exercise was unclear [21]. Consequently, exercise-based CR was considered cost-saving compared to CR without exercise, and an effective secondary prevention strategy in reducing subsequent cardiac events and re-admissions, and increased survival [21]. Comparatively, other studies did not find significant evidence identifying any interventions as conclusively cost-effective, and this is likely due to inappropriate use of time horizons, perspective, choice of health outcomes, or cost categories.

Nevertheless, cost-effectiveness results can accurately be non-conclusive. Only two evaluations performed subgroup analysis, finding that interventions were more cost-effective depending on gender and risk of disease progression [2]. Given expected differences in cost and health effects for patients of different gender, ages, disease severity, and comorbidities, subgroup analysis is recommended to explore heterogeneity of results between relevant patient groups.

When analysing cost-effectiveness estimates for CR evaluations, it is key to consider input uncertainty on results, and observe whether statistical significance or minimally important differences are achieved. Presenting only deterministic results can be misleading and may show the intervention to be highly cost-effective, yet closer scrutiny of the confidence intervals in some cases reveals very limited certainty around the result [4]. Findings should therefore be reported showing deterministic results of the base case as well as subgroup analyses and measures of uncertainty such as confidence intervals and/or (probabilistic) sensitivity analyses. These will provide a fair representation of findings, statistical significance, achievement of MID, and the potential effect of unknowns on the decision to be made.

These findings provide the basis for the following recommendations for future economic evaluations of CR programs:

1. Include comorbid patients.
2. Use of longer time-horizons (ideally lifetime) to capture the long-term health and cost-related outcomes of exercise-based CR for chronic cardiovascular-related conditions.
3. Develop an effective standardised exercise-based CR program to enhance comparability of health outcomes between studies.
4. Develop standardised cost categories consistent with the study perspective to enhance comparability of economic findings between studies, potentially including relevant non-health care costs such as productivity loss.
5. Adhere to up-to-date standards for economic evaluations of healthcare interventions.
6. Use subgroup analysis to capture the effects of exercise-based CR on different patient groups.
7. Use standardised reporting guidelines (e.g. CHEERS) to enhance study comparability.
8. Report confidence intervals, outcome measures and MIDs to enhance the quality of methodological reporting.

Crucially, following the above recommendations will allow carers and providers to make better-informed choices about the CR programs most suitable for their specific patient groups or setting, as the particulars of each will bring specific value weights to the various elements of the costs and outcomes associated to specific modalities of CR programs.

Limitations of this review include incomplete retrieval of all economic evaluations of exercise-based CR-services, which may have arisen from the exclusion of some electronic or grey literature sources. As most economic evaluations are published or cited in economic and scientific journals, it is likely these effects will be minimal following an extensive literature search of several online databases.

## Conclusion

Evidence exists supporting the cost-effectiveness of exercise-based CR for cardiovascular disease patients. Variability between studies in study perspective, time horizon, setting, comparators, included costs and interventions makes it difficult to compare and assess cost-effectiveness between alternative strategies. Future studies may wish to consider the implications of an exercise-based CR program for patients with comorbidities and employ longer time-horizons. This will allow the long-term effects of CR services to be better understood and in a majority patient group that presents to this pathway. Standardisation of CR service and delivery will enable greater comparability between studies on a clinical and cost level, with the program providing maximum patient-provider benefit to be identified. Future economic evaluations of exercise-based CR should adhere to current guidelines for the reporting of healthcare interventions. The methodology of cost-effectiveness evaluations could be further improved to accommodate different standards and processes between countries.

## Appendix 1

### Electronic search strategy: MEDLINE (OVID)

1. cost
2. costs
3. cost analysis
4. cost-analysis
5. cost analyses
6. cost-analyses
7. cost benefit
8. cost-benefit
9. cost benefit analysis
10. cost-benefit analysis
11. cost benefit analyses
12. cost-benefit analyses
13. cost effective
14. cost-effective
15. cost effectiveness
16. cost-effectiveness
17. cost effectiveness analysis
18. cost-effectiveness analysis
19. cost effectiveness analyses
20. cost-effectiveness analyses
21. cost minimisation
22. cost-minimisation
23. cost minimization
24. cost-minimization
25. cost minimisation analysis
26. cost-minimisation analysis
27. cost minimisation analyses
28. cost-minimisation analyses
29. cost minimization analysis
30. cost-minimization analysis
31. cost minimization analyses
32. cost-minimization analyses
33. cost utility
34. cost-utility
35. cost utility analysis
36. cost-utility analysis
37. cost utility analyses
38. cost-utility analyses
39. economic evaluation
40. economic assessment
41. health economics
42. 1 or 2 or 3 or 4 or 5 or 6 or 7 or 8 or 9 or 10 or 11 or 12 or 13 or 14 or 15 or 16 or 17 or 18 or 19 or 20 or 21 or 22 or 23 or 24 or 25 or 26 or 27 or 28 or 29 or 30 or 31 or 32 or 33 or 34 or 35 or 36 or 37 or 38 or 39 or 40 or 41
43. cardiac rehabilitation
44. 42 and 43

## Appendix 2

**Table 4:** Descriptive characteristics of included studies

Author (Year)	Study Design (time horizon)	Perspective	Setting	Location	Sample Size	Males (%) Cases/ Controls	Mean Age Cases/ Controls	Target Population	Subgroups	Co-morbidity	Comparators
Levin et al. (1991) [13]	Economic study of a 2-group non- randomized design (5 years)	Societal	Hospital-based	Sweden	305	84.4/84.8	57.3/57.2	MI patients	None	Not reported	Comprehensive CR vs standard care
Oldridge et al. (1993) [4]	Economic evaluation of a 2-group RCT (12 months)	Healthcare system + Patient	Not reported	Canada	201	88/89	52.9/52.7	AMI patients with mild to moderate depression or anxiety	None	Not reported	CR vs usual care
Ades et al. (1997) [16]	Retrospective economic evaluation on published data from randomized trials (15 years)	Patient or Payer	Majority hospital-based	USA	NA	100^a^	Majority <65^a^	AMI patients	None	Not reported	CR vs no CR
Carlson et al. (2000) [18]	Economic evaluation of a 2-group RCT (6 months)	Insurer	Rehabilitation centre	USA	80	81.6/83.3	59/59	Low risk cardiac patients	None	Not reported	Traditional vs Modified protocol
Hall et al. (2002) [15]	Economic evaluation of a 2-group RCT (12 months)	Healthcare system + Patient	Hospital-based	Australia	142	59/56	56/56	AMI patients	None	Not reported	REHAB vs ERNA (Early return to normal activities)
Southard et al. (2003) [9]	2-group RCT (6 months)	Healthcare system	Home-based (internet)	USA	104	68/82	61.8/62.8	Cardiovascular disease patients	None	Not reported	Home-based (internet) CR vs usual care
Yu et al. (2004) [19]	Economic evaluation of a prospective 2-group RCT (24 months)	Provider + Patient	Rehabilitation centre	Hong Kong	269	76/75	64/64	AMI or PCI patients	None	Not reported	CRPP vs conventional therapy
Briffa et al. (2005) [12]	Economic evaluation of an open RCT (12 months)	Healthcare system	Hospital-based	Australia	113	71.9/75	61.9/60.8	AMI or recovery from unstable angina	None	285 out of 2712 (11%) patients ineligible due to comorbidity	Comprehensive CR vs conventional care
Reid et al. (2005) [17]	Economic evaluation of a 2-group RCT (24 months)	Healthcare system	Rehabilitation Centre	Canada	392	85/84	58/58	CAD (AMI, PCI, CABG and angina) patients.	None	Co-morbid conditions that may impair progress were addressed (i.e. depression, musculoskeletal/respiratory problems).	CR (3-month) vs CR (12-month)
Jolly et al. (2007) [11]	Economic evaluation of a 2-group RCT (24 months)	Societal	Hospital-based	UK	525	77.2/76.0	60.3/61.8	MI or revascularization (PTCA/CABG) patients (within 12 weeks)	None	219 out of 1997 (11%) patients ineligible due to comorbidity Some patients adapted program to suit their co-morbidities.	Home vs Hospital
Taylor et al. (2007) [10]	Economic evaluation of a 2-group RCT (9 months)	Healthcare system + Patient	Hospital-based	UK	104	NR	NR	AMI patients	None	Patients with a major co-morbidity were excluded.	Home vs Hospital
Dendale et al. (2008) [14]	Retrospective economic evaluation of a non-randomised clinical trial (4.5 years)	Healthcare Payer	Hospital-based	Belgium	213	75.9/66.3	58.6/64.8	PCI patients	None	Patients with life-threatening or symptomatic co-morbidities were excluded.	CR vs no CR
Huang et al. (2008) [21]	Retrospective economic evaluation based on the United States Renal Data System (42 months)	Insurer	Not reported	USA	4324	72^a^	Majority >65^a^	ESRD (CABG) patients	Age, gender, number of cardiovascular conditions, diagnosis of chronic obstructive pulmonary disease, race, Medicaid coverage, higher serum albumin, primary diagnosis of diabetes, AMI before CABG and propensity for CR	Documented the number of existing cardiovascular conditions at initiation of dialysis.	CR vs no CR
Papadakis et al. (2008) [2]	Economic evaluation of a 2-group RCT (24 months)	Healthcare system	Rehabilitation Centre	Canada	392	84.5/89.3	58.4/58.4	CAD (MI,PCI,CABG and angina) patients	Cardiac risk level, risk of disease progression, reason for referral and sex.	Not reported	CR (3-month) vs CR (12-month)
Spronk et al. (2008) [20]	Economic evaluation and modelling of CR strategies (Lifetime)	Societal	Not reported	USA	NA	100^a^	64^a^	CAD (MI) patients.	None	Not reported	CR only vs revascularisation before CR or after CR failure

RCT = randomised controlled trial, MI/AMI = acute myocardial infarction, PCI/PCTA = percutaneous coronary intervention, ESRD = end stage renal disease, CABG = coronary artery bypass grafting, CR =cardiac rehabilitation ^a^For modelling and cohort studies, single figures given reflect the demographic characteristics of the overall study population, where in remaining trials these statistics are given for each intervention arm

## Appendix 3

**Table 5:** Descriptions of the interventions of included studies

Study	REHAB Arm	Program Duration	Total Number of Sessions (Duration)	Education Component						Exercise Component (FITT)				
				Education	Diet	Smoking	Counselling	Relaxation/ Stress Management	Behaviour Change	Exercise	Frequency	Intensity	Time	Type
Levin et al. (1991) [13]	Comprehensive CR Initial Training	24 months 3 months		Yes	Yes^c^	Yes^c^	Yes^c^			Yes	Twice weekly	Patient HR(max) – 5 beats^c^	45 mins	Cycling, jogging and calisthenics.
	Standard care			No						No				
Oldridge et al. (1993) [4]	CR	8 weeks	16 sessionso	Unclear			Yes (Group sessions, 12 h)^d^			Yes	Twice weekly^d^	65% of Patient HR(max)^d^	50 mins^d^	Low-level exercise prescription
	Usual care									Unclear				
Carlson et al. (2000) [18]	Traditional protocol Weeks 1–4	6 months 1 month		Yes	Yes (3 sessions)		Yes			Yes	Thrice weekly	Patient prescribed HR range, 60–85% capacity	30–40 min	Warm up, aerobic exercise with continuous ECG monitoring and a cool down
	Weeks 5–12	2 months		Yes						Yes	Thrice weekly	Patient prescribed HR range, 60–85% capacity	30–40 min	Warm up, aerobic exercise with continuous ECG monitoring and a cool down
	Weeks 13–25	3 months		Yes						Yes	Thrice weekly	Patient documented	Patient documented	Maintenance program.
	Modified protocol Weeks 1–4	6 months 1 month		Yes	Yes (3 sessions)					Yes	Thrice weekly	Patient prescribed HR range, 60–85% capacity	30–40 min	Warm up, aerobic exercise with continuous ECG monitoring and a cool down
	Weeks 5–12	2 months	Yes	Yes (support/education meetings, weekly week 6 > ~1 h)				Yes	Bandura’s self-efficacy theory	Yes	Twice weekly (week 6>)			
	Weeks 13–25	3 months		Yes (support/education meetings, weekly ~1 h)	Yes			Yes	Bandura’s self-efficacy theory	Yes	Once weekly (weeks 11–17) Once bi-weekly (weeks 18–25)			
Hall et al. (2002) [15]	REHAB	6 weeks	14 sessions (average attendance)	Yes	Yes					Yes	Up to 4 days per week	NR	NR	Low level training program and home walking program
	ERNA	2 weeks		Yes			Yes			Yes				Home walking program
Southard et al. (2003) [9]	Special intervention (internet)	6 months	NA	Yes	Yes					Yes	Patient dependent	Patient dependent	Patient dependent	Patient dependent
	Usual care									Unclear				
Yu et al. (2004) [19]	CRPP (Phase I)	7–14 days												Inpatient ambulation program
	(Phase II)	8 weeks	16 sessions (Each session, 3 h)	Yes (Each session, 1 h)	Yes	Yes		Yes		Yes	Twice weekly	65–85% age adjusted HR reserve	Each session, 2 h	Aerobic cardiotraining including treadmill, ergometry, rower, stepper, arm ergometry, and dumbbell and weight training.
	(Phase III)	6 months												Community-based home exercise program
	(Phase IV)	24 months												Maintenance program
	Conventional therapy			Yes (2 h talk)						No				
Briffa et al. (2005) [12]	Comprehensive outpatient CR	6 weeks	18 sessions	Yes (9 h)	Yes		Yes (4.5 h)	Yes		Yes	Thrice weekly	NR	Each session, 1–1.5 h	Aerobic circuit training and resistance training
	Conventional therapy					Yes		Unclear						
Reid et al. (2005) [17]	Standard CR	3 months	33 sessions	Yes (Total, 6 h)	Yes	Yes	Yes	Yes	Yes	Yes	Twice weekly (27 sessions)	Resting HR with 50–80% reserve	1 h	The frequency, intensity, duration and mode of exercise were consistent with guidelines for CAD patients.
	Distributed CR	12 months	33 sessions	Yes (Total, 6 h)	Yes	Yes	Yes	Yes	Yes	Yes	Tapered (27 sessions)	Resting HR with 50–80% reserve	1 h	The frequency, intensity, duration and mode of exercise were consistent with guidelines for CAD patients.
Jolly et al. (2007) [11]	Hospital-based (Hospital 1)	12 weeks	24 sessions (up to 3 h)	Yes (Each session, optional)		No	Yes	Yes (Each session, voluntary)		Yes	Twice weekly	60–75% max HR		Walking, built up to 25–30 min of fixed cycling, rowing
	(Hospital 2)	9 weeks	9 sessions (1.5 h)	Yes (Weekly)	Yes (Total, 40 mins)	No	Yes	Yes (Each session)		Yes	Once weekly	NR		Circuit training with six stations (1–2 min per station) and additional walking.
	(Hospital 3)	8 weeks	12 sessions (8 sessions × 2.5 h)	Yes (8 sessions)	Yes (Total, 30 mins)	Yes (Total, 30 mins)	Yes	Yes (Weekly)		Yes	Twice weekly	65–75% max HR		45 mins of circuit training
		(4 sessions × 1 h)								Yes	Once weekly	65–75% max HR	1 h	45 mins of circuit training
	(Hospital 4)	6 weeks	12 sessions 8 sessions (2 h)	Yes (Each session, 30 mins)	Yes (Total, 30 mins)	No	Yes	Yes (Each session)		Yes	Twice weekly	65–75% max HR		Warm up, then 40 mins of exercise on fixed bikes and treadmills
		4 sessions (1 h)	No								Once weekly	65–75% max HR	1 h	Warm up, then 40 mins of exercise on fixed bikes and treadmills
	Home-based	6 weeks	NA	Yes		No	Yes	Yes		Yes	Recommended daily			*Heart Manual* Home exercises, working up to daily walking and other physical activity
Taylor et al. (2007) [10]	Hospital-based	8–10 weeks	8–10 sessions (Each session, 2 h)	Yes^b^	Yes^b^		Yes^b^	Yes^b^		Yes	Once weekly	NR	NR	Aerobic exercise^b^
	Home-based	6 weeks	NA	Yes^b^				Yes^b^		Yes	Patient dependent	Patient dependent	Patient dependent	*Heart Manual* Aerobic exercise^b^
Dendale et al. (2008) [14]	CR	3 months		Yes	Yes	Yes (8 sessions)	Yes			Yes	Thrice weekly (at least 24 sessions)	Patient dependent; trained near ventilator threshold	~1 h	20 min of treadmill exercise, 20 min of cycling and 10 min of arm cranking
	No CR			Yes	Yes (8 sessions)	Yes	Yes			No				
Papadakis et al. (2007) [2]	Standard CR	3 months	33 sessions	Yes (6 h) ^a^	Yes^a^	Yes^a^	Yes^a^	Yes^a^		Yes	Twice weekly (27 sessions)	Resting HR with 50–80% reserve^a^	1 h^a^	The frequency, intensity, duration and mode of exercise were consistent with guidelines for CAD patients^a^
	Distributed CR	12 months	33 sessions	Yes (6 h)^a^	Yes^a^	Yes^a^	Yes^a^	Yes^a^	Yes	Yes	Tapered (27 sessions)	Resting HR with 50–80% reserve^a^	1 h^a^	The frequency, intensity, duration and mode of exercise were consistent with guidelines for CAD patients^a^

^a^As reported in a previous publication ^[17]^

^b^As reported in a previous publication ^[24]^

^c^As reported in a previous publication ^[23]^

^d^As reported in a previous publication ^[22]^

## Appendix 4

**Table 6:** Health-related data for included studies

Author (Year)	Health Outcomes	Follow up frequency and extent of Health outcomes	Patient sample loss to follow up	Effectiveness Measure (Instrument)	Preference-based Outcome
Levin et al. (1991) [13]	Total cost during study period (cost-benefit analysis)	NR	82/147 (56%) of patients completed initial 3-months. 78/147 (53%) of patients completed year-1. 72/147 (49%) of patients completed year-2. Note: figures given of CRPP arm	Total cost per patient	Not a preference-based outcome
Oldridge et al. (1993) [4]	HRQL	Baseline, 4, 8 and 12-months	NR	QALY (NR)	Time-trade off change score
Ades et al. (1997) [16]	Survival	N/A	N/A	Year of life saved	Not a preference-based outcome
Carlson et al. (2000) [18]	Cardiovascular health	Baseline and 6-months	At 6-months, 67/80 (84%)	Primary: maximal oxygen consumption, low-density lipoprotein cholesterol	Not a preference-based outcome
Hall et al. (2002) [15]	HRQL	Every week for 6-weeks, then 3, 6 and 12-months.	13 (9%) patients did not complete any questionnaires (adjusted sample size, *n* = 129) At 3-months, 98/129 (76%) patient data At 6-months, 101/129 (78%) patient data At 12-months, 76/129 (59%) patient data	Return to normal activities and Quality of Life score (Health Measurement Questionnaire)	Not a preference-based outcome
Southard et al. (2003) [9]	Monetary equivalent for (costs of) cardiovascular events	Baseline and 6-months	At 6-months, 100 (96%) patient data	Health care costs	Not a preference-based outcome
Yu et al. (2004) [19]	HRQL	Phase 1 (baseline), phase 2, 3 and 4 (2-years)	At 2-yrs., data available for 204 (76%) patients.	QALY (SF-36)	Patient reported time trade off score
Briffa et al. (2005) [12]	HRQL	Baseline, 6 and 12-months	At 6-months, 109/113 (96%) patient data At 12-months, 105/111 (95%) patient data	QALY (SF-36 and UBQ-H)	Preference-based utility based on UBQ-H questionnaire
Reid et al. (2005) [17]	Exercise, cardiac risk, HRQL, depression	Baseline, 3, 12 and 24-months	At 3-months, 344 (87.8%) patient data At 12-months, 314 (80.1%) patient data At 24-months, 252 (64.2%) patient data	Peak oxygen uptake, kilojoules of energy related to activity, HDL-C and TGs, cardiac events, Heart diseases HRQL (McNew instrument), generic HRQL (SF-36), Depressive symptoms (Centre for Epidemiological Studies Depression scale)	Not a preference-based outcome
Jolly et al. (2007) [11]	HRQL	6,12 and 24-months	At 6-months, 485 (93%) patient data At 12-months, 475 (91.5%) patient data At 24-months, 461 (89%) patient data	QALY (EQ-5D)	EQ-5D summary score based on UK value set tariff
Taylor et al. (2007) [10]	HRQL	Baseline, 3 and 9-months	At 9-months, data available for 48 (80%) and 32 (73%) of the home- and hospital-based groups respectively.	QALY (EQ-5D)	EQ-5D summary score based on UK value set tariff
Dendale et al. (2008) [14]	Monetary equivalent for (costs of) cardiovascular events	Two examinations in 6-month period, single examination annually thereafter	NR	Health care costs	Not a preference-based outcome
Huang et al. (2008) [21]	Survival	N/A	N/A	Year of life saved	Not a preference-based outcome
Papadakis et al. (2007) [2]	HRQL	3, 6, 12, 15 and 24-months.	At 2-yrs., data available for 307 (78%) patients.	QALY (NR)	Time trade off preference-based utility scores
Spronk et al. (2008) [20]	HRQL	N/A	N/A	QALY	Time-trade off preference-based utility score

HRQL = Health related quality of life, NR = not reported, N/A = not applicable

## Appendix 5

**Table 7:** Economic data for included studies

Author (Year)	Resource Use	Source of Costs	Non-medical costs	Characterising Uncertainty	Characterising Heterogeneity	Currency (Price, Year)	Findings
Levin et al. (1991) [13]	For direct costs, the source of data on resource use is unclear. For indirect costs, resource loss data (e.g. sick leave) is gathered from the Swedish National Health Insurance system.	For indirect cost, production loss calculated using data from The Swedish Health Insurance System (NHIS).	Productivity loss due to patient sick leave/ early retirement and time cost of training/ outpatient visits.	Sensitivity analysis	Did not perform subgroup analysis	Swedish Kroner (SEK), 1988 Costs based on an exchange rate of £1 = SEK10.90, September 1990. Costs discounted at a rate of 5%.	Total direct costs: CR group (40, 240 SEK), no CR group (44,150 SEK); Difference (3910 SEK) Total costs (including patient time costs and loss of production): CR group (484,260 SEK) no CR group (55,7770 SEK); Difference (73,510 SEK)
Oldridge et al. (1993) [4]	Data on healthcare service utilisation was taken from patients following the intervention (e.g. number of visits to physicians, emergency departments, other health departments and community cardiac rehabilitation programs)	Staff salaries based on Ontario Health Insurance Plan and local wage rates. Program costs estimated per patient as the sum of the costs of renting space, equipment, staff salaries, printed resource literature and patient parking expenses.	Patient travel, time, equipment and childcare cost	Scenario analysis	Did not perform subgroup analysis	USD$ Costs expressed as 1991 USD$ based on Canadian/US currency exchange rates and US medical care inflation.	Best estimate incremental cost $480 (range $230–$1280). QALY gained with CR: 0.052.
Ades et al. (1997) [16]	Reference to previous study in which offset savings attributable to rehospitalisation were calculated from a computerised review of billing data from five regional hospitals.	Data derived from published results of randomised trials.	Not included	Sensitivity analysis	Did not perform subgroup analysis	USD$, 1988 Costs projected to 1995 using the US Consumer Price Index for inflation Years per life saved, YLS projections based on annual discount rate of 5%.	Net cost of CR: $430 ($1280–850). (Discounted) incremental life expectancy: 0.202 years Cost effectiveness value of $2130/YLS (1980s), projected to $4950/YLS for 1995
Carlson et al. (2000) [18]	Unclear	Staff costs based on full-time equivalents	Not included	Not Reported	Did not perform subgroup analysis	USD$?	Cost difference: MP $738 less/patient than TP.
Hall et al. (2002) [15]	Patient groups assumed to have the same resource requirements for in-hospital treatment. Use of non-hospital services by patient self-administered questionnaire. Use of hospital services (hospitalisations) collected from medical records.	Program cost calculated from unit cost data taken from Westmead Hospital.	Patient travel costs	Not Reported	Did not perform subgroup analysis	AUD$, 1999	CR program cost: $21.57 per session per patient Est. total program cost (14-sessions) $393.68 (includes estimate for hospital overheads of $91.70) The net cost saved by targeting CR to high-risk patients approx. $300 per low-risk patient.
Southard et al. (2003) [9]	Resource use data (e.g. hospitalisations and emergency room visits) identified from patient report.	1-week time analysis conducted to calculate staff time. UB92 forms containing exact cost data was available for nine patients. For one patient cost data was estimated from AHRQ Healthcare Cost & Utilization Project using ICD-9 codes and the 1999 National Data Set.	Not included (program cost incorporated a $30 patient subscription)	Not Reported	Did not perform subgroup analysis	USD$? 2001?	Total expenditures for major cardiovascular events: intervention group ($31,110), usual care ($104,684); Gross cost savings ($1418 per person) Fewer cardiovascular-related events: intervention group (15.7%), usual care group (4.1%)
Yu et al. (2004) [19]	Resource use data collected during trial; patient reported direct medical expenses when private practitioners were consulted.	Healthcare costs based on published hospital costs. Costs of hospitalisations, investigations, interventions, clinic visits taken from local official publication on hospital charges. Drug costs on published local drug formulary.	Not included	Not Reported	Did not perform subgroup analyses	USD$ Dates of unit costs not reported	Direct healthcare cost: CR Group ($15,292), no CR Group ($15,707); Difference ($415) The 2-yr. mean gain in QALYs: CR Group (0.6) The cost utility ratio for the CR program: -$650
